# Commentary: Comment on “Comparative analysis of blood routine, C-reactive protein, and biochemical markers in children with *Mycoplasma pneumoniae* pneumonia and its coinfections”

**DOI:** 10.3389/fped.2026.1756271

**Published:** 2026-05-29

**Authors:** Zhijun Jiang, Yuchang Fei, ZhiWei Hu

**Affiliations:** 1 Jiashan County Maternity and Child Health Care Hospital, Jiaxing, Zhejiang, China; 2 Department of Integrated Chinese and Western Medicine, The First People’s Hospital of Jiashan, Jiashan Hospital Affiliated of Jiaxing University, Jiaxing, Zhejiang, China; 3 Department of Endocrinology, The First People’s Hospital of Jiashan, Jiashan Hospital Affiliated of Jiaxing University, Jiaxing, Zhejiang, China

**Keywords:** blood routine, *Haemophilus influenzae*, influenza virus, *Mycoplasma pneumoniae*, pediatric pneumonia

Dear Editor,

We read with great interest the article entitled “Comparative analysis of blood routine, C-reactive protein, and biochemical markers in children with *Mycoplasma pneumoniae pneumonia* and its coinfections” by Tang et al. (1). Their retrospective analysis addresses a clinically relevant topic and provides valuable insignts for optimizing the diagnosis and management of pneumonia in children. However, two critical limitations regarding diagnostic validity and severity assessment require clarification to enhance the study's scientific rigor.

## Insufficient validation of *Mycoplasma pneumoniae* (MP) diagnostic methods

The study confirmed *Mycoplasma pneumoniae pneumonia* (MPP) using immunoglobulin (Ig)-M (titer ≥1:160) and RNA isothermal amplification. However, it lacks key details needed to ensure diagnostic accuracy. First, the study did not report the agreement between serological and molecular tests, which is a critical omission given their inconsistent performance in children. The United States (U.S.) Centers for Disease Control and Prevention (CDC) explicitly notes that serological testing for MP lacks specificity and often requires paired acute and convalescent sera to minimize false negatives, as single-sample IgM detection may miss early infections due to delayed seroconversion (2). Second, single MP-IgM testing shows poor sensitivity and specificity in acute pediatric MPP. In a study of 144 confirmed cases, sensitivity was 85.5%, but specificity was only 25.6%, indicating it is unreliable as a standalone diagnostic too (3). Another study demonstrated MP-IgM sensitivity as low as 61.2%, meaning around 38.8% of children with true MP infection would be falsely negative (4). Without details on the timing of testing relative to symptom onset or the agreement between tests, the validity of the “MPP-confirmed” cohort is questionable.

## Lack of standardized pneumonia severity assessment

The conclusion that *Haemophilus influenzae* coinfection worsens disease severity lacks support from standardized evaluations. A meta-analysis indicates that severity assessment in pediatric pneumonia is hindered by inconsistent definitions and lack of consensus on core indicators (5). Most studies rely on non-standardized outcomes, such as length of hospital stay, which do not reflect the disease's biological characteristics. Moreover, this review emphasizes that effective severity assessment requires integrating objective clinical indicators, such as oxygenation status and respiratory rate, instead of relying solely on symptom duration. The British Thoracic Society (BTS) guideline for pediatric community-acquired pneumonia (CAP) delineates severity-based criteria to inform clinical decision-making; however, Ambroggio et al. (6) reported in their study that this guideline exhibits suboptimal sensitivity, specifically with regard to the evaluation of need for hospitalization and the determination of patient disposition. Additionally, hospital length of stay correlates weakly with true disease severity because it is influenced by non-clinical factors such as parental requests and bed availability. Therefore, objective indicators like oxygen saturation and the need for respiratory support are essential for robust severity stratification. Omitting validated scales and objective metrics weakens the evidence supporting a causal relationship between coinfection and disease severity.

## Suggestions for improvement

We propose the following suggestions to further enhance the scientific rigor and clinical value of the study:

1. It is recommended to supplement key diagnostic validation data. Report concordance rates between MP-IgM (titer ≥1:160) and RNA isothermal amplification. Additionally, specify the interval from symptom onset to testing for each patient. If feasible, validate the “MPP-confirmed” cohort with paired acute and convalescent sera by following CDC guidelines to reduce false negatives in diagnosis of MP in children.
2. For standardized pneumonia severity assessment, we suggest re-analyzing the association between coinfection and severity using the World Health Organization (WHO) Pediatric Pneumonia Severity Scale or the modified British Thoracic Society (BTS) criteria. Incorporate objective indicators such as oxygen saturation, need for respiratory support, and extent of chest CT consolidation. Use these to replace or complement hospital length of stay, which may be influenced by non-clinical factors. Also, further verification is suggested to confirm whether *Haemophilus influenzae* coinfection correlates with aggravated disease severity.

We sincerely appreciate the authors for their valuable contribution to understanding *Mycoplasma pneumoniae pneumonia* and its coinfections in children. Implementing the above recommendations will further enhance the study's validity and impact, benefiting clinical practice and future research.
